# Lung tumor presenting with acute myocardial infarction and lower extremity arterial embolism

**DOI:** 10.1186/s12872-020-01770-0

**Published:** 2020-11-11

**Authors:** Jichun Liu, Hao Chen, Xiangrong Xie, Yuwen Yang, Shengxing Tang

**Affiliations:** 1 Department of Cardiology, The First Affiliated Hospital of Wannan Medical College (Yijishan Hospital of Wannan Medical College), Wuhu, 241001 Anhui People’s Republic of China; 2 Department of Pathology, Wannan Medical College, Wuhu, 241002 Anhui People’s Republic of China

**Keywords:** Acute myocardial infarction, Lung tumor, Arterial embolism, Case report

## Abstract

**Background:**

Lung tumor embolization leading to acute myocardial infarction (AMI) is rare. Previouscases of lung tumor embolization were reported in the coronary artery. We describe here a case of lung tumor embolization leading to the simultaneous occurrence of AMI and lower extremity arterial embolism.

**Case presentation:**

A 64-year-old patient was admitted to the emergency department complaining of chest pain and was diagnosed with AMI.An echocardiography showed a mass in the left atrium that was speculated to be a myxoma. An emergency coronary angiography found no evidence of atherosclerosis. On the second day of admission, the patient was diagnosed with lower extremity arterial embolism. Initially, we speculated that the left atrium myxoma caused an embolism resulting in the AMI and lower extremity arterial embolism.However, a lung tumor was the real cause of both conditions. Unfortunately, the patient abandoned treatment when he learned of his disease and died three days later after being discharged from the hospital.

**Conclusions:**

Lung tumor embolism is an extremely rare cause of AMI. Even rarer is the case presented here, in which a lung tumor embolism caused AMI and lower extremity arterial embolism. Clinicians should recognize lung tumor embolism as a potential cause of AMI.

## Background

Acute myocardial infarction(AMI) is usually caused by plaque rupture, erosion, ornodules in the coronary arteries, although other uncommon etiologies exist [1]. Thromboembolism is a common complication in cancer patients. According to pre-existing reports, 4.5% of patients develop venous thromboembolism, and 1.5% of patients develop arterial thromboembolism; these conditions may result from the hypercoagulable state associated with cancers. Acute arterial embolismdue to tumor embolus is a rare complication in cancer patients [2], even rarer is lung tumor embolization leading to AMI. Lung tumor embolization was previously reported to occur in the coronary artery [3–5]. Here, we report a case of lung cancer embolus leading to the simultaneous occurrence of AMI and lower extremity arterial embolism.

## Case presentation

A 64-year-old non-smoking man with no hypertension or diabetes was admitted to the emergency department complaining of oppressive chest pain accompanied by profuse sweating lasting for four hours upon admission. His temperature was 36.1 °C, respiratory rate: 22 breaths/minute, pulse rate: 81 beats/minute, blood pressure: 114/68 mmHg, and finger oxygen saturation was 100% on ambient air. His rapid troponin I levels were 4.83 ng/ml.

**Fig.1:**
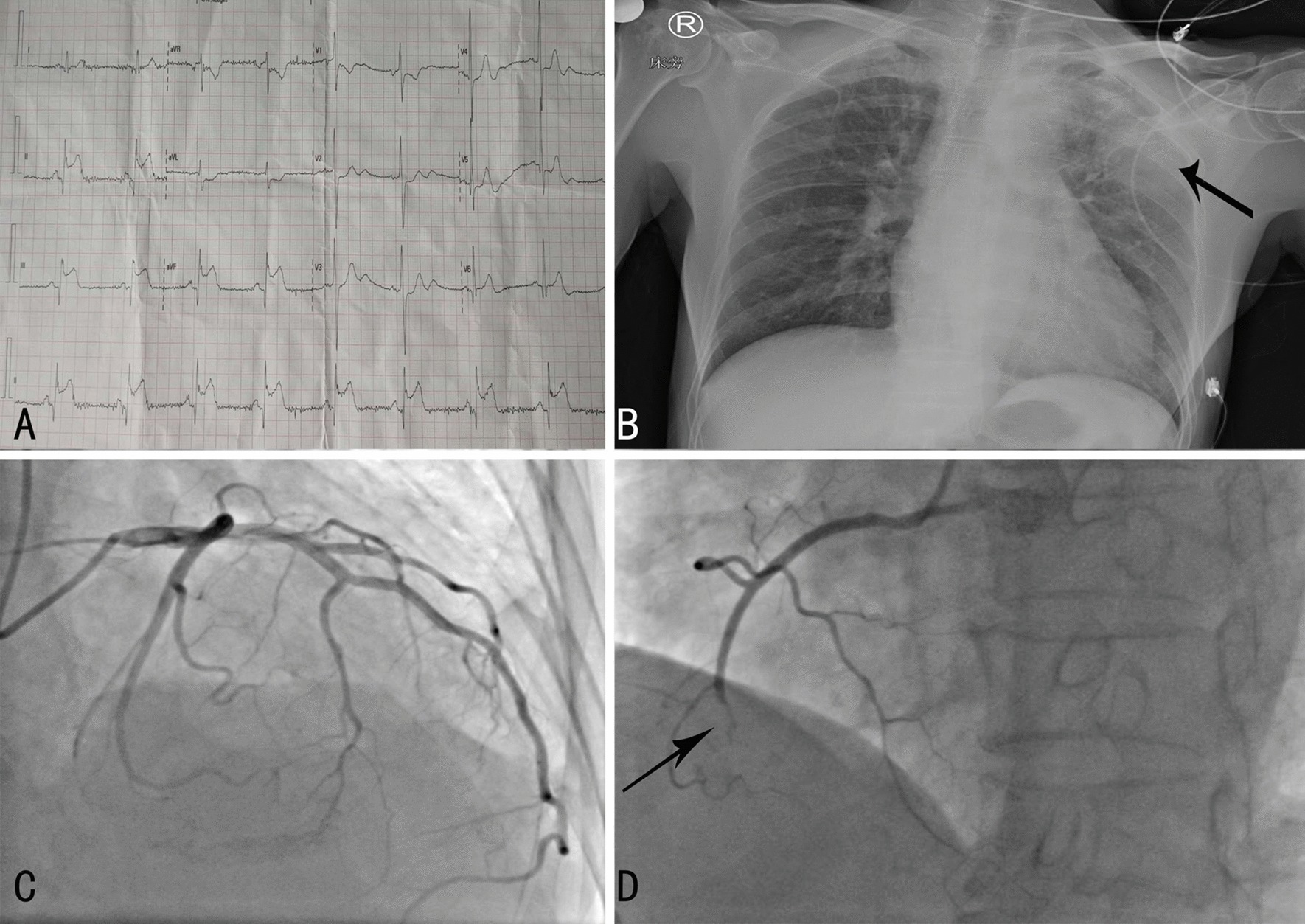
Results of the electrocardiogram, bedside posteroanterior chest X-ray, and coronary angiography. **a** The electrocardiogram showed normal sinus rhythm and ST-segment elevations in leads II, III and aVF. **b** A bedside posteroanterior chest X-ray revealedsuspected pneumonia in the upper left lung(black arrow). **c** A coronary angiography found abnormalities in the left coronary artery. **d** The middle-to-distal right coronary artery was completely occluded(black arrow)

An electrocardiogram revealed normal sinus rhythm and ST-segment elevations in leads II, III, and aVF(Fig. 1a).A bedside echocardiography revealed abnormal movement in the left inferior ventricular wall and a mass in the left atrium, speculated to be a myxoma. A bedside posteroanterior chest X-ray showed suspected pneumonia in the upper left lung(Fig. 1b). The patient was diagnosed with AMI. An emergency coronary angiography showed no evidence of atherosclerosis, but did reveal that the middle-to-distal right coronary artery was completely occluded (Fig. 1d), indicating an embolism without an acute manifestation of atherosclerosis.

Despite intracoronary injection of tirofiban, aspiration, and balloon dilation, blood flow was not recovered at the distal end of the right coronary artery, and no thrombus was extracted. After the percutaneous coronary intervention, antiplatelet, lipid regulation and anticoagulant therapy were provided for the patient.

**Fig.2:**
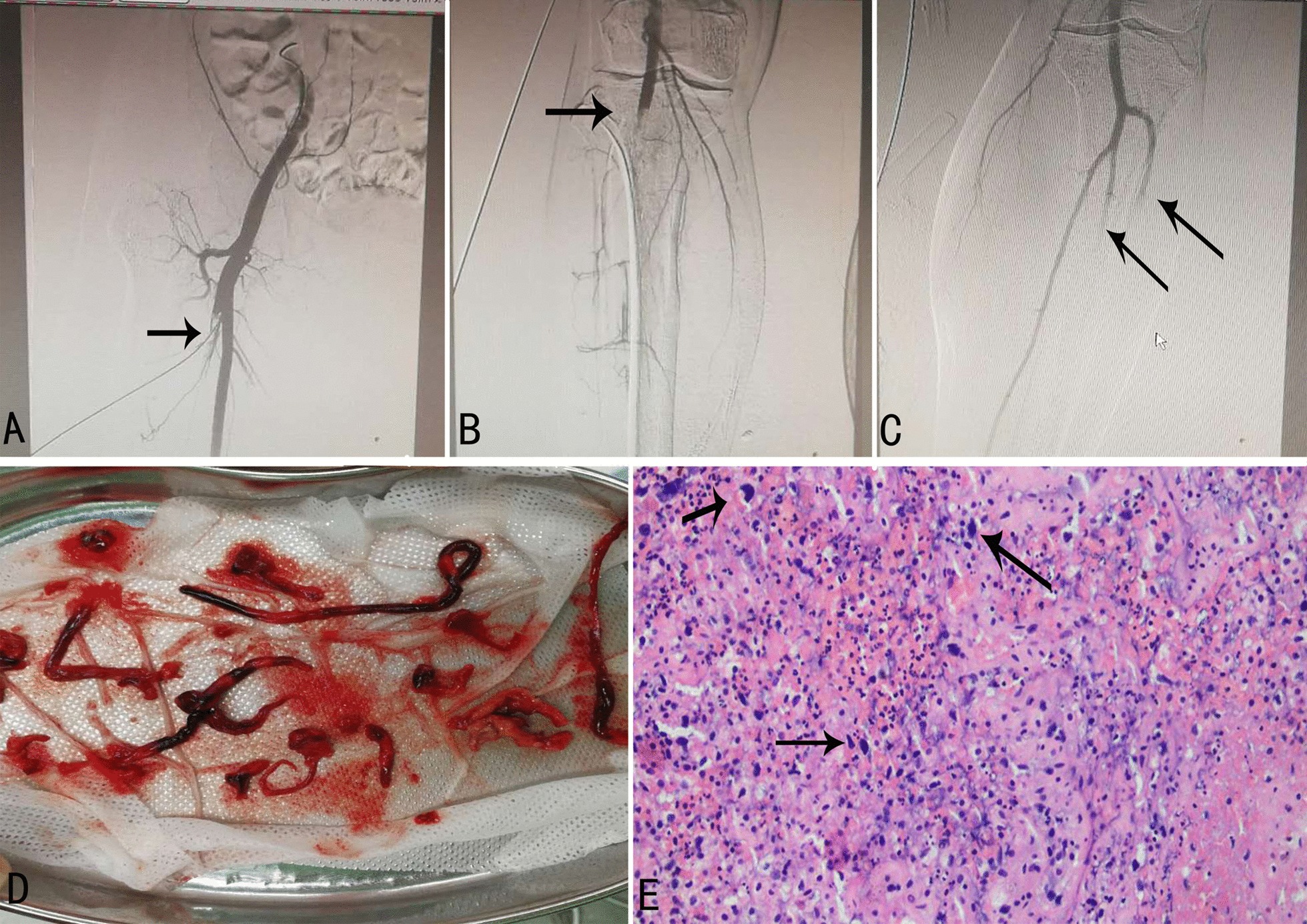
Results from angiography of lower limb arteries and lower extremity tissue as well as pathological examination of tissue and thrombus. **a** The onset of the right profunda artery was occluded(black arrow). **b** Occlusion of the right popliteal artery (black arrow). **c** The distal left posterior tibial and peroneal arteries were completely occluded(black arrow). **d** Lower extremity tissue and thrombus. **e** Coagulation necrosis with atypia cells(H&E stain, 200 × , black arrow)

On the second day of admission, the patient began to suffer from pain in both lower limbs, which was accompanied by low skin temperature and hemoptysis. Bedside Color Doppler ultrasound of the lower extremities showed incomplete embolization of the popliteal and anterior tibial arteries of the lower right extremities. Emergency angiography of lower limb arteries revealed that the onset of the right profunda artery and the popliteal artery were completely occluded, as were the distal left posterior tibial and peroneal arteries(Fig. 2a–c).

**Fig.3:**
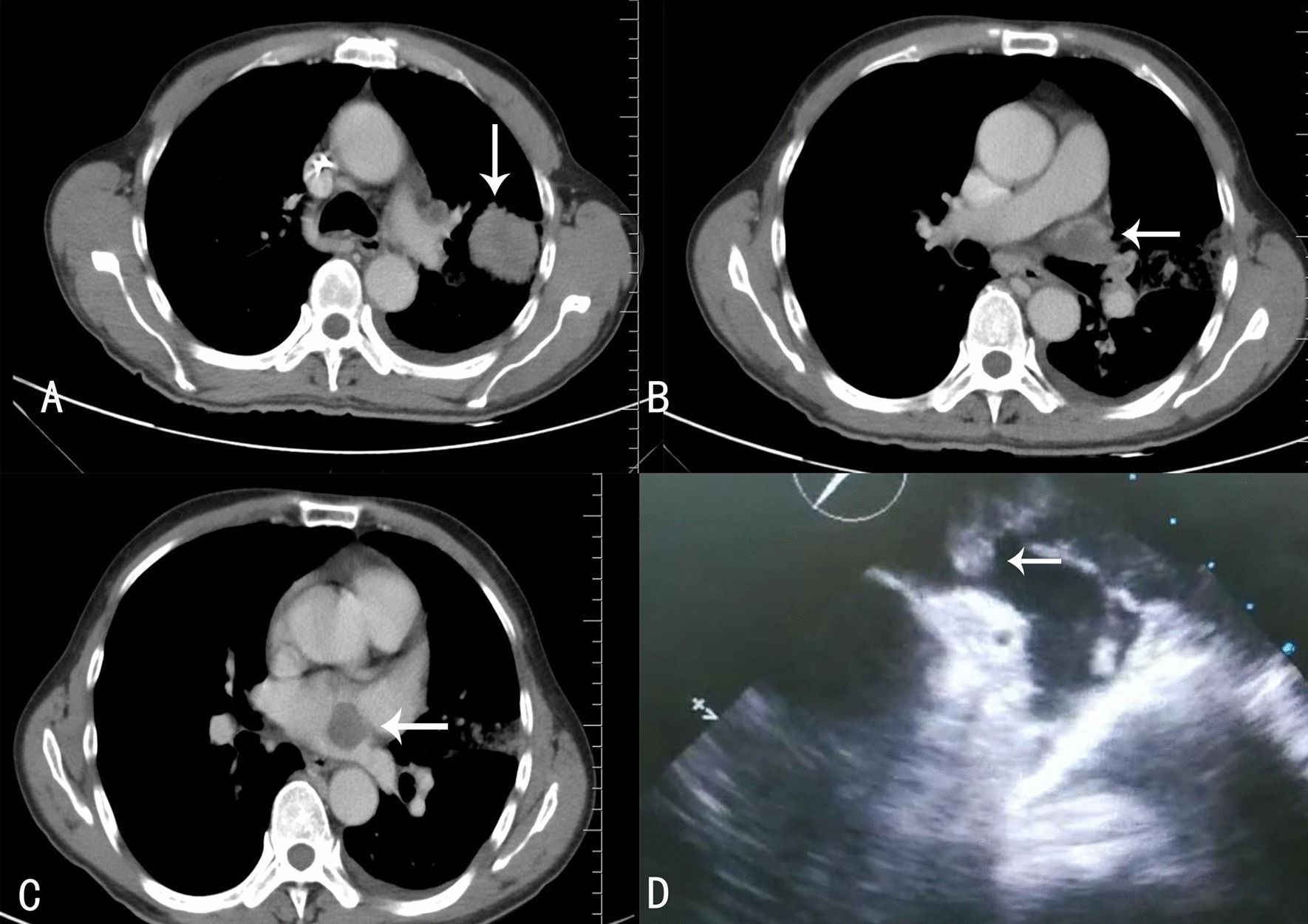
Chest computed tomography (CT) scan and transesophageal echocardiography. **a** Solid lesion with irregular borders in the upper left lobe of the lung (white arrow). **b** The upper left pulmonary vein was occluded by solid material(white arrow). **c** Solid mass in the left atrium(white arrow). **d** Transesophageal echocardiography revealed a solid mass in the left atrium growing from the entrance of the left superior pulmonary vein (white arrow)

To prevent ischemic necrosis of the lower extremities, a lower extremity arteriotomy was performed under general anesthesia, and a large amount of tissue and thrombus were removed. Due to hemoptysis, afiberoptic bronchoscopy was performed under general anesthesia, and the results showed the presence of a large blood clot in the left bronchus and its subsequent airways. To discern the growth location of the left atrial mass, we also performed a transesophageal echocardiography, which showed that the mass in the left atrium grew from the entrance of the left superior pulmonary vein(Fig. 3d).

**Fig.4:**
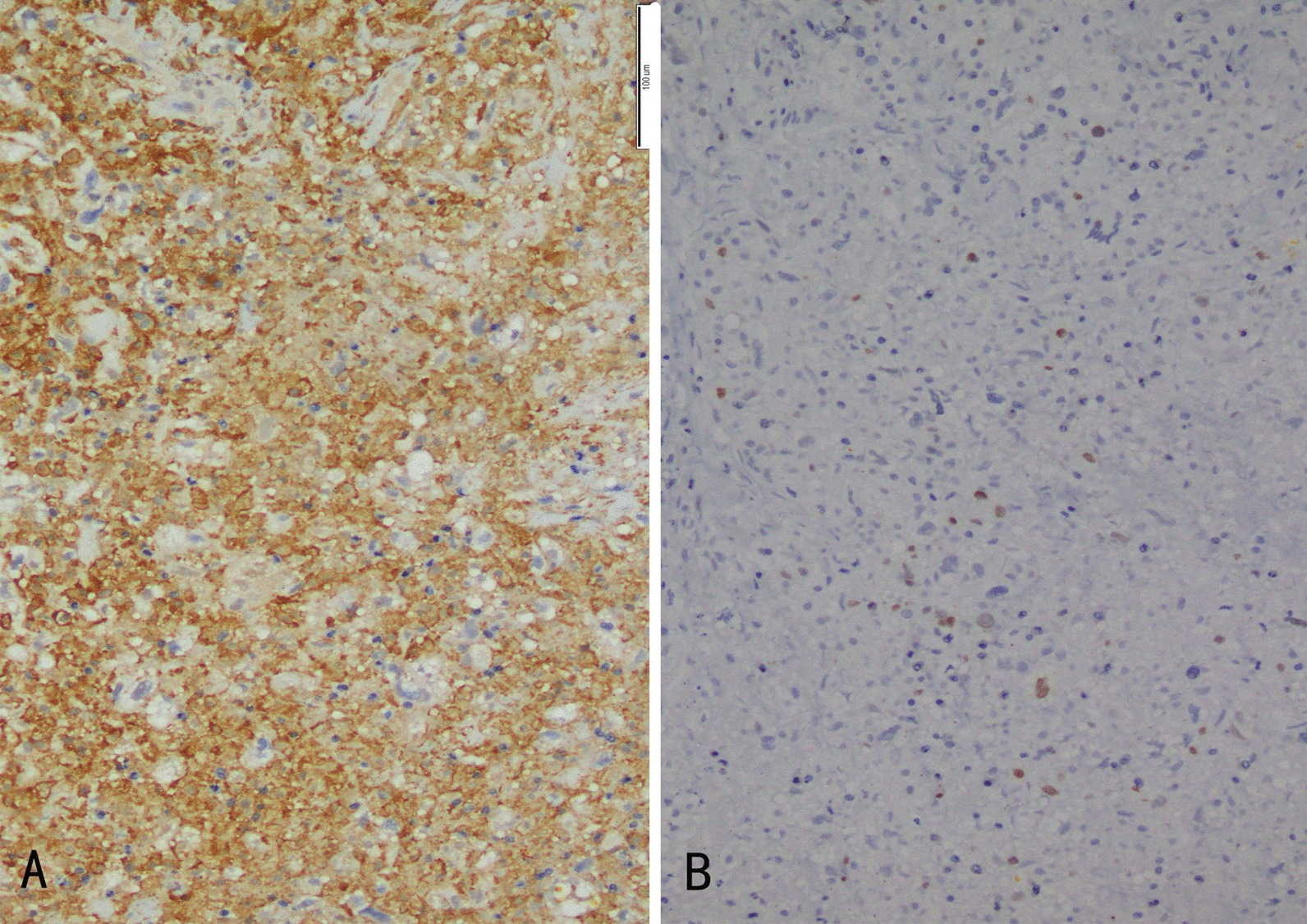
Tissue and thrombus histology. **a** The tissue and thrombusshowed positive immunostainigfor CD163 (200 ×). **b** The proliferation index (Ki-67) was approximately 2% (immunostaining, 200 ×)

Subsequent pathological examination of the tissue and thrombususing H&E staining revealed coagulation necrosis with atypia cells (Fig. 2e). Immunohistochemical staining was positive for CD163(Fig. 4a) and negative for SMA, CD31, ERG, CK7, TTF, NapsinA, CK5/6, P63, and P40. The proliferation index (Ki-67) was approximately 2% (Fig. 4b).

A chest computed tomography (CT) scan revealed a solid lesion of the upper left lobe of the lung with irregular borders, speculated to be a lung tumor (Stage IVA). The tumor seemed to enter into the left atrium via the upper left pulmonary vein, which appeared completely occluded due to the presence of solid material in its lumen(Fig. 3a–c). Based on the patient’s medical history and examination, tumor embolism was suspected. Unfortunately, when he learned of his disease, the patient abandoned treatment and was discharged from the hospital. A week later, the patient's family was contacted by phone, and they disclosed that the patient had died three days after he was discharged.

## Discussion and conclusions

Coronary artery embolism(CE), in which obstructive material enters the coronary artery, block its blood flow, and cause ischemia, is an uncommon cause of AMI. The prevalence of AMI resulting from CE is 2.9%. AMI resulting from CE can be caused by left atrial myxoma, atrial fibrillation, cardiomyopathy, valvular heart disease, malignancy, infective endocarditis, and atrial septal defects. According to the currently available data, no significant difference exists in the incidence of CE between the left anterior descending branch, left circumfluence branch, and right coronary artery [6].

The most common sources of arterial tumor emboli originate from primary lung tumors [7]. Generally, lung tumors can spread to the heart through hematogenous dissemination, the lymphatic system, direct invasion, and by intracavitary diffusion via the inferior vena cava or pulmonary veins. Lung tumors spread to the heart most commonly via thelymphatic system rather than through the pulmonary veins. The entry of lung tumors into left atrium via the pulmonary vein is uncommon [8]. Acute arterial embolism caused by tumor embolus is a rare complication in cancer patients.The accepted mechanism of arterial embolism in malignant lung tumors is that the tumor nests are expelled from the system after invading the pulmonary veins [2]. AMI caused by lung tumor embolus has been reported [5, 9]. Lung tumor embolizationcan also occur in other arterial systems including the aorta, the femoral artery, arteries of the extremities, and the mesenteric artery [7]. In this case, thrombus aspiration was performed in the coronary artery, but the thrombus could not be removed. We hypothesize that the cause was a small hole on the side of the suction catheter which was unable to extract larger tumor emboli.

Tumor embolism is an extremely rare cause of AMI. Even rarer is the case reported here, in which tumor embolismlead to AMI and arterial embolism of the lower extremities. However, clinicians should recognize that tumor embolismis a potential cause of AMI.

## Data Availability

The data analyzed in this case report are not publicly available due to the privacy policies of the hospital but may be requested from the correspondingauthor if deemed reasonable.

## Abbreviations

AMI: Acute myocardial infarction
CE: Coronary artery embolism
CT: Computed tomography
